# Supplementary material for: Ocean and land forcing of the record-breaking Dust Bowl heatwaves across central United States
Source: Nat Commun. 2020 Jun 8;11:2870. doi: 10.1038/s41467-020-16676-w (PMC7280240; doi:10.1038/s41467-020-16676-w)
Supplement: Supplementary file 1 — Supplementary Information [file 41467_2020_16676_MOESM1_ESM.pdf]

Supplementary information and figures for

Ocean and land forcing of the record-breaking Dust Bowl heatwaves across central United States

by Cowan[1,2,3] et al.

[1]*Centre for Applied Climate Sciences, University of Southern Queensland, Toowoomba, Queensland, Australia.*

[2]*Bureau of Meteorology, Melbourne, Victoria, Australia.*

[3]*School of GeoSciences, University of Edinburgh, Edinburgh, United Kingdom.*

Supplementary Table 1: List of the Coupled Model Intercomparison Project Phase 5 models utilized in this study. Models from the same institute have been grouped together.

**Model acronym**	**Model name**
ACCESS1-0 | ACCESS1-3	Australian Community Climate and Earth-System Simulator, version 1.0 | version 1.3
bcc-csm1-1 | bcc-csm1-1-m	Beijing Climate Center, Climate System Model, version 1.1 | 1.1 moderate resolution
BNU-ESM	Beijing Normal University Earth System Model
CanESM2	Second Generation Canadian Earth System Model
CCSM4	Community Climate System Model, version 4
CESM1-BGC	Community Earth System Model, version 1
CMCC-CMS	Centro Euro-Mediterraneo sui Cambiamenti Climatici Stratosphere-resolving Climate Model
CNRM-CM5	Centre National de Recherches Météorologiques Coupled Global Climate Model, version 5
CSIRO-Mk3-6-0	Commonwealth Scientific and Industrial Research Organisation Mark 3.6.0
FGOALS-s2	Flexible Global Ocean–Atmosphere–Land System Model, second spectral version
GFDL-ESM2G | GFDL-ESM2M | GFDL-CM3	Geophysical Fluid Dynamics Laboratory Earth System Model with Generalized Ocean Layer Dynamics (GOLD) component

	∣ Earth System Model with Modular Ocean Model 4 (MOM4) component ∣ Climate Model, version 3
inmcm4	Institute of Numerical Mathematics Coupled Model, version 4.0
IPSL-CM5A-LR ∣ IPSL-CM5A-MR	L’Institut Pierre-Simon Laplace Coupled Model, version 5A coupled with Nucleus for European Modelling of the Ocean (NEMO), low resolution ∣ mid resolution
MIROC5 ∣ MIROC-ESM	Model for Interdisciplinary Research on Climate, version 5 ∣ Earth System Model
MPI-ESM-LR ∣ MPI-ESM-MR ∣ MPI-ESM-P	Max Planck Institute Earth System Model, low resolution ∣ medium resolution ∣ paleoclimate
MRI-CGCM3	Meteorological Research Institute Coupled Atmosphere–Ocean General Circulation Model, version 3
NorESM1-M	Norwegian Earth System Model, version 1, intermediate resolution

Supplementary Table 2: Center year of the decade where record-breaking heatwave frequency values occur across the largest area (% in brackets) of the central US (105°W-85°W, 30°N-50°N) for each Coupled Model Intercomparison Project Phase 5 preindustrial control simulation century. Years listed are each model's own years. Underlined events are those removed from the ensemble analysis due to possible contamination from the same underlying decadal sea surface temperature pattern. The four models marked with an asterisk are not used in the historical ensemble (Fig. 1a, Supplementary Fig. 1). Observations are from the Berkeley Earth Surface Temperature (BEST) dataset and the Global Historical Climatology Network-Daily (GHCN-D) archive.

			**Model years**		
**Model**	Years 1-100	Years 101-200	Years 201-300	Years 301-400	Years 401-500
ACCESS1-0	399 (22)	**401 (22)**	592 (39)	631 (31)	708 (54)
ACCESS1-3	260 (37)	366 (40)	511 (49)	619 (29)	657 (55)
bcc-csm1-1	40 (60)	199 (70)	**203 (55)**	345 (40)	464 (26)
bcc-csm1-1-m*	81 (27)	**105 (42)**	300 (17)	**326 (31)**	- (-)
CanESM2	2030 (21)	2154 (43)	2289 (28)	2353 (17)	2503 (40)
CESM1-BGC	191 (31)	294 (31)	392 (43)	482 (36)	562 (38)
CMCC-CMS	3766 (32)	3831 (84)	3960 (40)	4058 (27)	4131 (34)
CNRM-CM5	1908 (23)	1950 (39)	2149 (20)	2203 (61)	2277 (23)
CSIRO-Mk3-6-0	15 (31)	158 (42)	278 (18)	379 (28)	440 (33)
BNU-ESM*	1522 (17)	1598 (34)	1673 (43)	1752 (47)	1912 (26)
FGOALS-s2*	1924 (25)	1982 (59)	2088 (23)	2217 (24)	2266 (16)
GFDL-ESM2G	72 (26)	189 (41)	291 (54)	330 (37)	449 (51)
GFDL-ESM2M	83 (33)	138 (49)	207 (26)	375 (25)	435 (20)
inmcm4*	1856 (33)	1953 (29)	2123 (28)	2213 (25)	2295 (25)
IPSL-CM5A-LR	1865 (51)	1938 (35)	2094 (53)	**2107 (28)**	2279 (39)

MIROC5*	2080 (34)	2174 (16)	2221 (29)	2325 (16)	2440 (31)
MIROC-ESM	1819 (23)	1961 (43)	2073 (30)	2109 (40)	2298 (30)
MPI-ESM-LR	1855 (34)	2032 (59)	**2056 (41)**	2211 (18)	2266 (23)
MPI-ESM-MR	1929 (48)	1981 (48)	2077 (39)	2168 (46)	2279 (38)
MPI-ESM-P	1866 (26)	1964 (36)	2129 (24)	2196 (37)	2344 (51)
MRI-CGCM3	1902 (33)	2025 (43)	2068 (38)	2156 (38)	2292 (19)
NorESM1-M	743 (29)	832 (24)	901 (43)	1073 (24)	1152 (33)
CCSM4	- (-)	- (-)	- (-)	- (-)	- (-)
IPSL-CM5A-MR	- (-)	- (-)	- (-)	- (-)	- (-)
GFDL-CM3	- (-)	- (-)	- (-)	- (-)	- (-)
Observations: GHCN-D | BEST	1935 (71) | 1935 (58)				

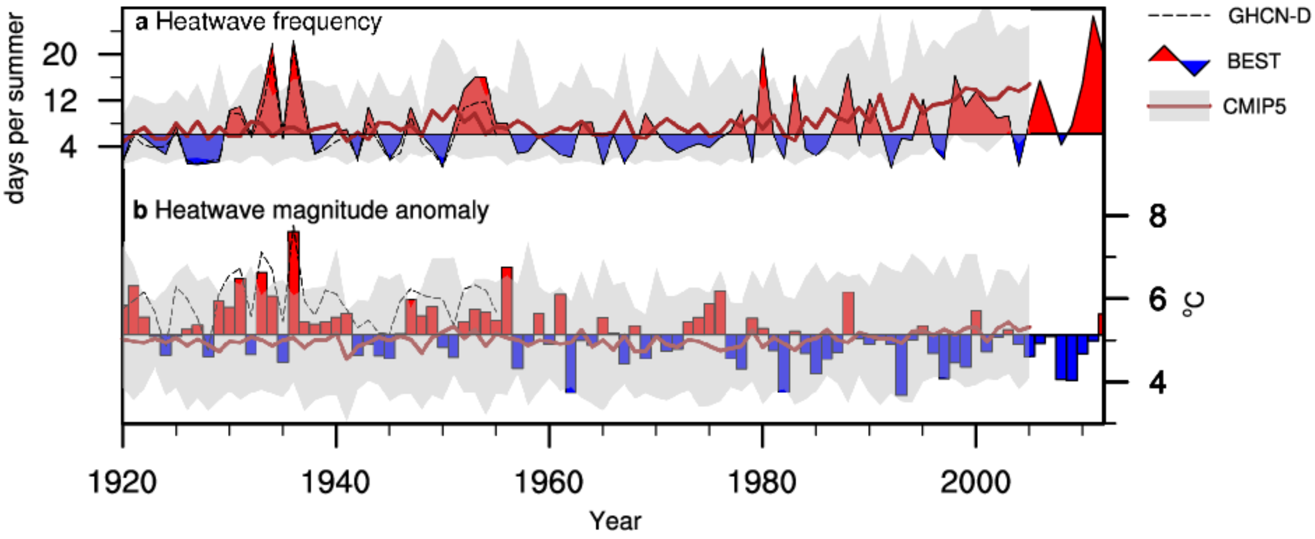

Supplementary Figure 1: Observed and simulated central US heatwave conditions. Time evolution of observed summer heatwave frequency (a) and magnitude (b) anomalies averaged over the central US (105°W-85°W, 30°N-50°N). Shown are gridded observations from the Berkeley Earth Surface Temperature (BEST) dataset (color) and Global Historical Climatology Network-Daily (GHCN-D) stations (dotted line; up to 1955), and a 20 member Coupled Model Intercomparison Project Phase 5 (CMIP5) historical model ensemble (grey line with shading representing the 10th – 90th percentile range). The climatology period is defined as 1920-2012 for observations and 1920-2005 for CMIP5.

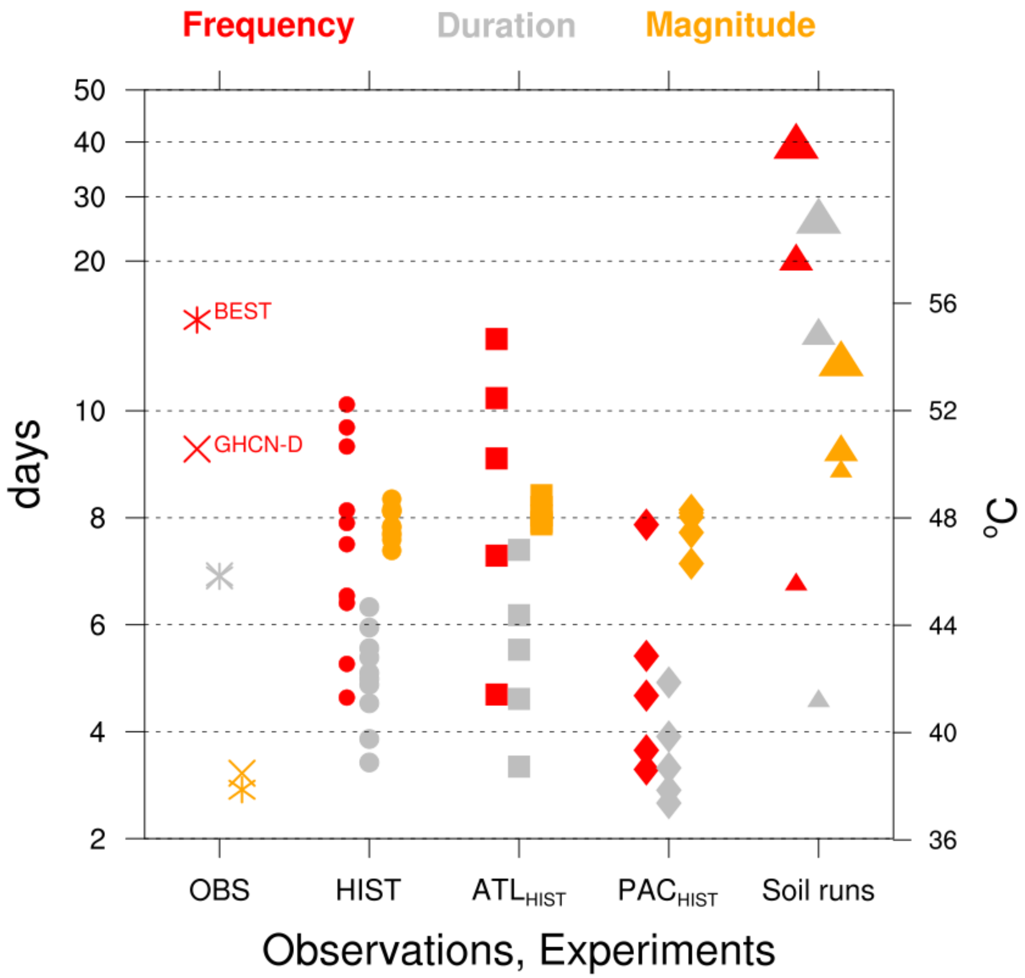

Supplementary Figure 2: Spread of simulated summer heatwave conditions during the Dust Bowl. Observed and simulated summer heatwave metrics averaged over 1930-37 for the southern-central US (105°W-85°W, 30°N-40°N). Shown are heatwave frequency and duration (red and grey, respectively; left axis), and magnitude (orange; right axis) for observations (OBS) and Hadley Centre Global Environment Model version 3 (HadGEM3) simulation ensembles forced with historical sea surface temperatures over all ocean basins (HIST; circles), the Atlantic only (ATL_{HIST}; squares) and the Pacific only (PAC_{HIST}; diamonds). The three idealized soil simulations (triangles) are the 30% (small), 50% (medium) and 80% soil run (large), while observations are from Global Historical Climatology Network-Daily (GHCN-D; crosses) stations and Berkeley Earth Surface Temperature (BEST; asterisk).

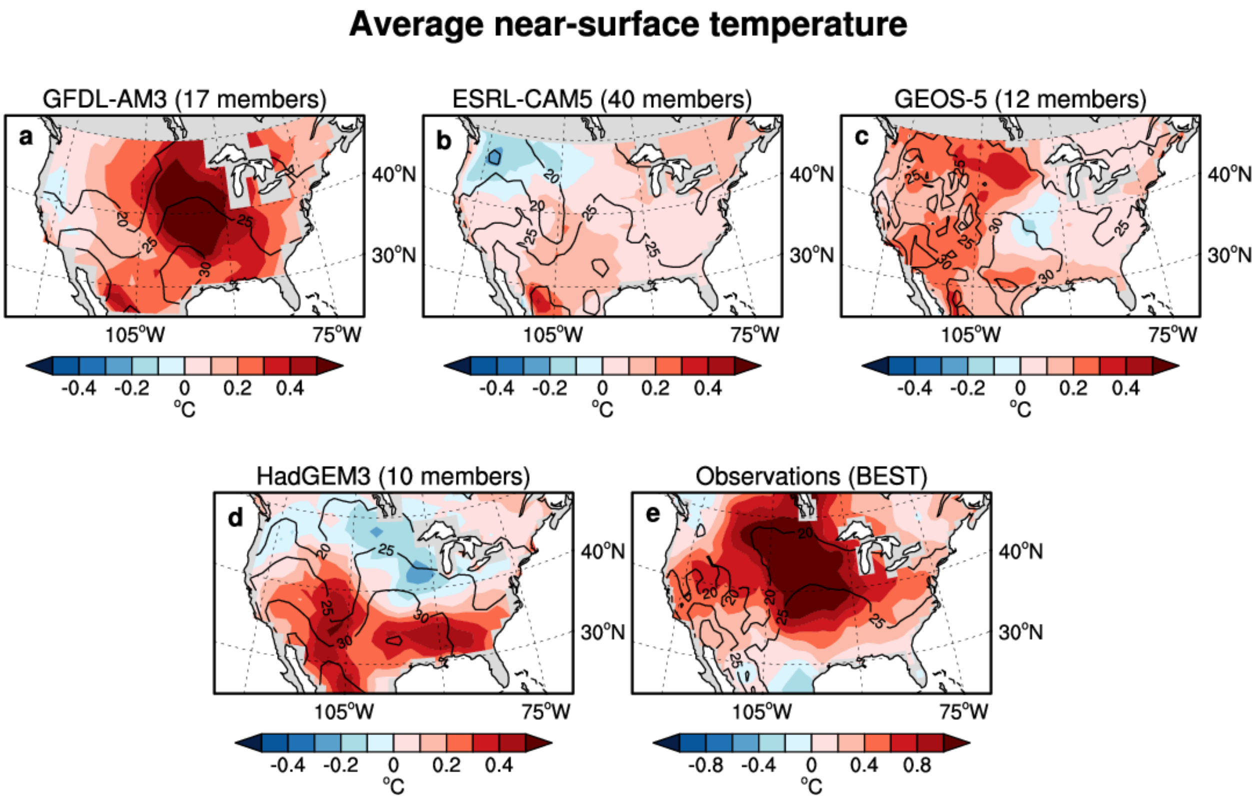

Supplementary Figure 3: Atmosphere-only general circulation model comparison for summer temperatures. Near-surface summer temperatures (contour lines) and anomalies (color) from four atmosphere-only general circulation models (AGCMs), averaged over 1930-37 and driven with observed sea surface temperatures, for ensemble means from Geophysical Fluid Dynamics Laboratory-Atmospheric Model version 3 (GFDL-AM3; a), Earth System Research Laboratory-Community Atmosphere Model version 5 (ESRL-CAM5; b), Goddard Earth Observing System, Version 5 (GEOS-5; c) and Hadley Centre Global Environment Model version 3 (HadGEM3; d). e, Observed pattern from the Berkeley Earth Surface Temperature (BEST) dataset, noting the anomalies are twice that of the AGCMs. All anomalies are referenced to a 1916-1955 climatology.

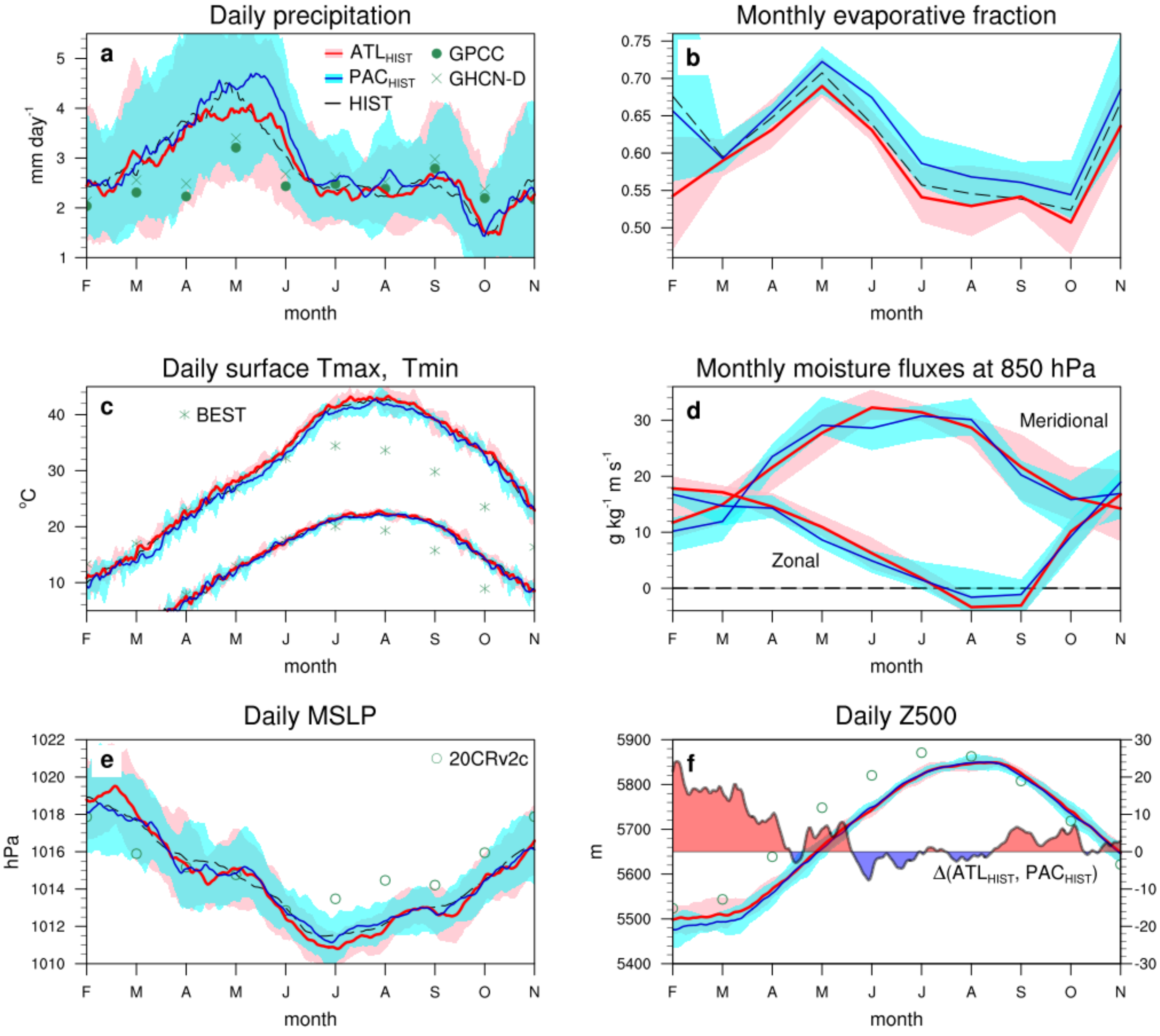

Supplementary Figure 4: Climatological conditions during the Dust Bowl. Observed and simulated climatologies averaged over 1930-37 for the southern-central US (105°-85°W, 30°-40°N), for daily precipitation (a), monthly evaporative fraction (b), daily surface maximum and minimum temperature (Tmax, Tmin) (c), and monthly 850 hPa zonal and meridional moisture fluxes (d). Observations are from the Global Precipitation Climatology Centre (GPCC; filled circles) dataset, Global Historical Climatology Network-Daily (GHCN-D; crosses) stations and

the Berkeley Earth Surface Temperature (BEST; asterisks) dataset, while simulations are Hadley Centre Global Environment Model version 3 (HadGEM3) ensembles forced with historical sea surface temperatures over all ocean basins (HIST; black dotted line), the Atlantic only (ATL_{HIST}; red line) and the Pacific only (PAC_{HIST}; blue line). The pink and cyan shading represents the ensemble range of the ATL_{HIST} and PAC_{HIST} simulations, respectively. Averaged over the Midwest to eastern US (105°-75°W, 30°-50°N) are simulated climatologies of daily mean sea level pressure (MSLP; e), and daily 500 hPa geopotential height (Z500; f), and monthly reanalysis from the Twentieth Century Reanalysis version 2c (20CRv2c; non-filled circles). Also shown in (f) is the difference (Δ) between the ATL_{HIST} and PAC_{HIST} ensembles (right vertical axis). Precipitation, MSLP and Z500 are all smoothed using a 15-day running mean. This figure suggests that differences in spring precipitation prior to the most active heatwave summers in the ATL_{HIST} and PAC_{HIST} ensembles stem from precipitation deficits in the ATL_{HIST} simulations in the southern-central US and anomalously wet conditions in the PAC_{HIST} simulations in April and May. This is consistent with a weakening of the meridional moisture transport from the Gulf of Mexico. For ATL_{HIST} in summer, this leads to a greater reduction in evaporative fraction, warmer maximum temperatures, and a deeper surface pressure low.

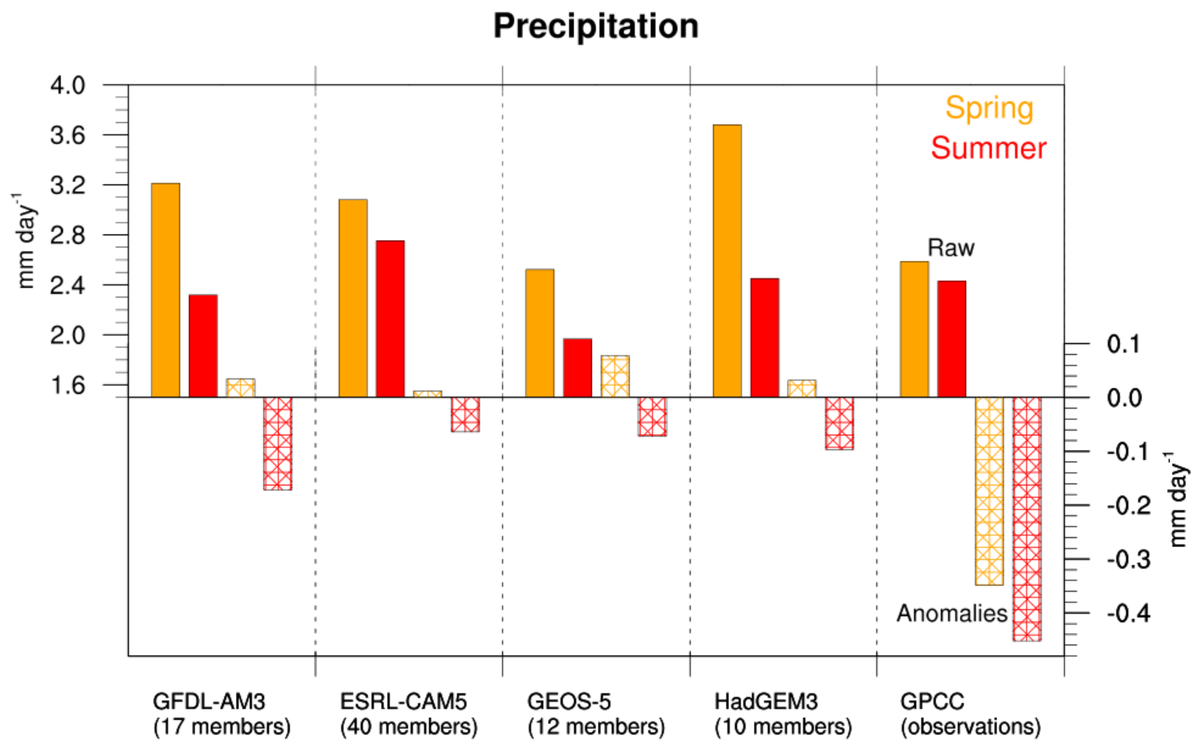

Supplementary Figure 5: Mean-state biases in precipitation over the southern-central US during the Dust Bowl. Raw and anomalous precipitation in four different atmosphere-only general circulation models (AGCMs) and observations from the Global Precipitation Climatology Centre (GPCC) over southern-central US (105°-85°W, 30°-40°N), composited over 1930-37. The AGCMs are Hadley Centre Global Environment Model version 3 (HadGEM3), Geophysical Fluid Dynamics Laboratory-Atmospheric Model version 3 (GFDL-AM3), Earth System Research Laboratory-Community Atmosphere Model version 5 (ESRL-CAM5) and Goddard Earth Observing System, Version 5 (GEOS-5). The number of ensemble members are shown in the parenthesis along the horizontal axes. The raw spring and summer conditions are shown as solid bars (left axes), while the anomalies are shown as hashed bars (right axis). Anomalies are calculated against each AGCM's 1916-1955 climatology.

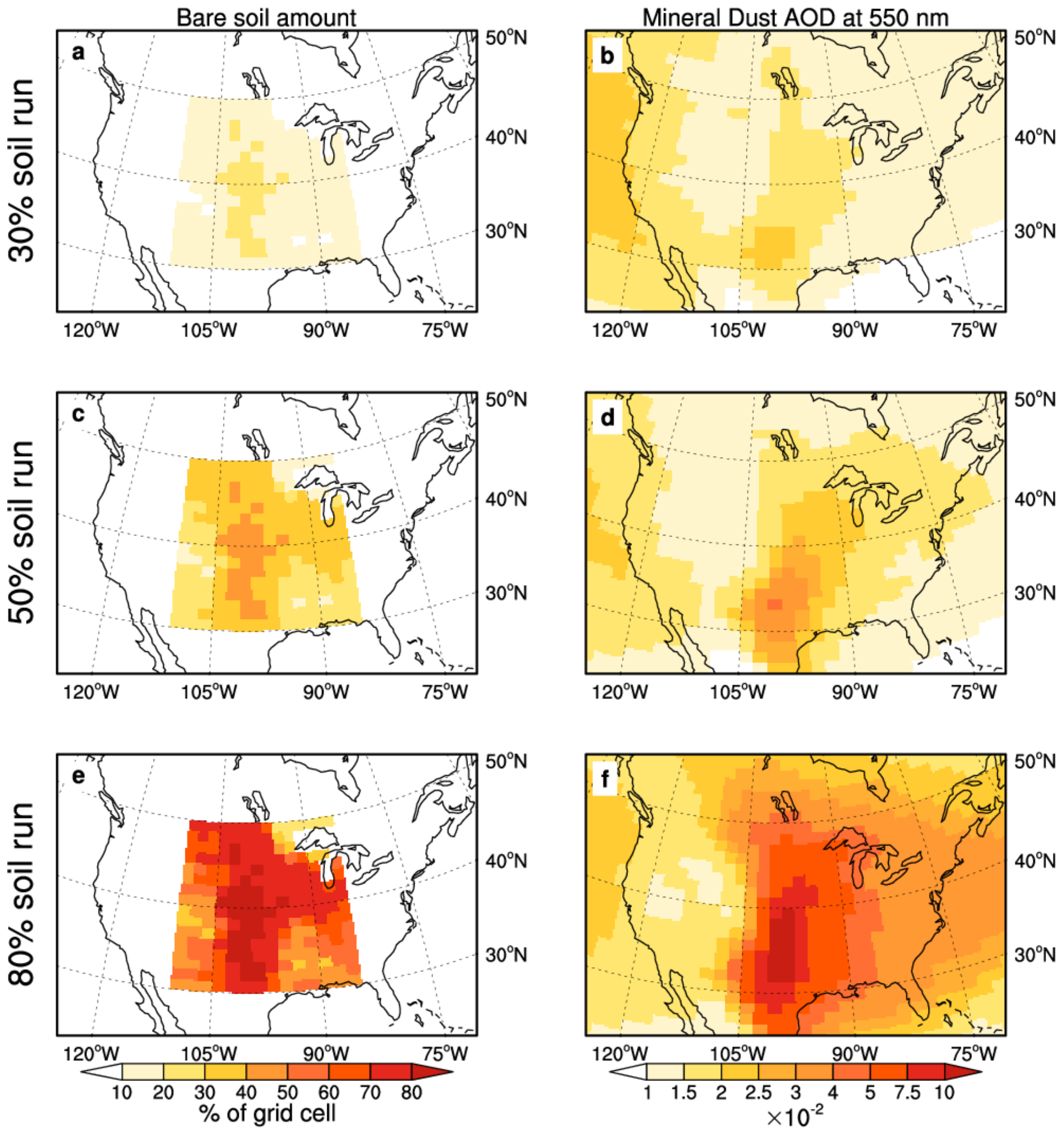

Supplementary Figure 6: Bare soil amounts and associated dust in bare soil simulations. Percentage of bare soil per grid cell over the central US (105°-85°W, 30°-50°N) in 1930, in the Hadley Centre Global Environment Model version 3 (HadGEM3) bare soil simulations where the regional average is 30% (a), 50% (c), and 80% (e)**.** The mean spring response in mineral dust Aerosol Optical Depth (AOD) at 550 nm averaged across 1928-1932, associated with the bare soil simulations is shown in (b,d,f) for the respective simulations. The percentage of bare soil is calculated as the amount of soil in the baseline historical sea surface temperature experiment plus

the amount converting the combined C3 and C4 grass fractions to bare soil. The bare soil experimental design is discussed in the study Methods section. Bare soil amounts outside the central US are not shown.

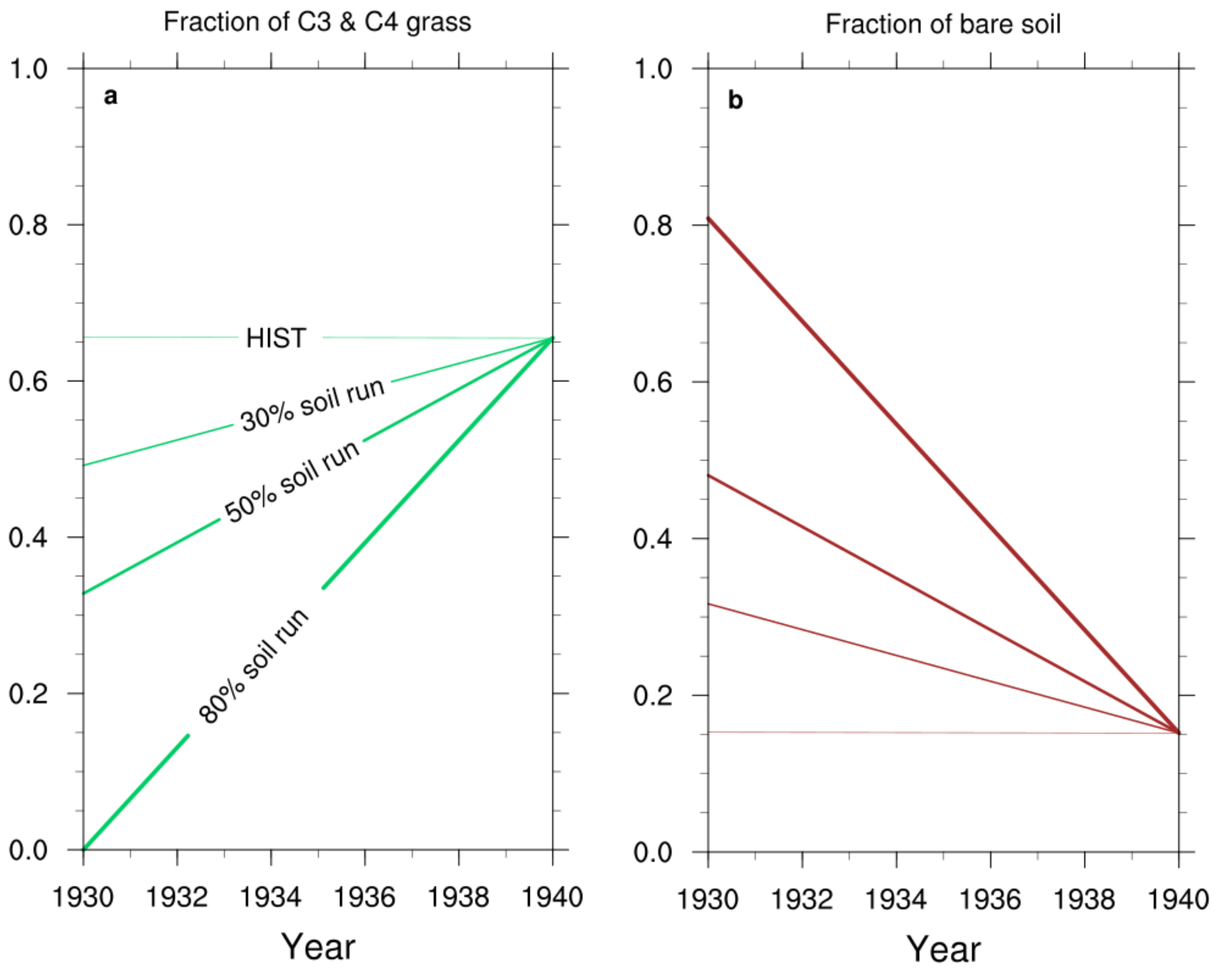

Supplementary Figure 7: Change in grass and bare soil fractions in the bare soil simulations. Fraction of C3 and C4 grass combined (a), and bare soil (b) within each land-surface grid-box, averaged over the central US (105°-85°W, 30°-50°N), for the four difference Hadley Centre Global Environment Model version 3 (HadGEM3) experiment types: historical sea surface temperature (HIST), 30% soil, 50% soil and 80% soil. Land-type fractions have a decadal temporal resolution in HadGEM3 and are linearly interpolated across decades, while the land surface changes preceding the Dust Bowl (increase in agriculture) and land degradation within the decade was very rapid[1]. We note here that the soil runs are highly idealized and are meant to highlight land-surface processes occurring during the Dust Bowl.

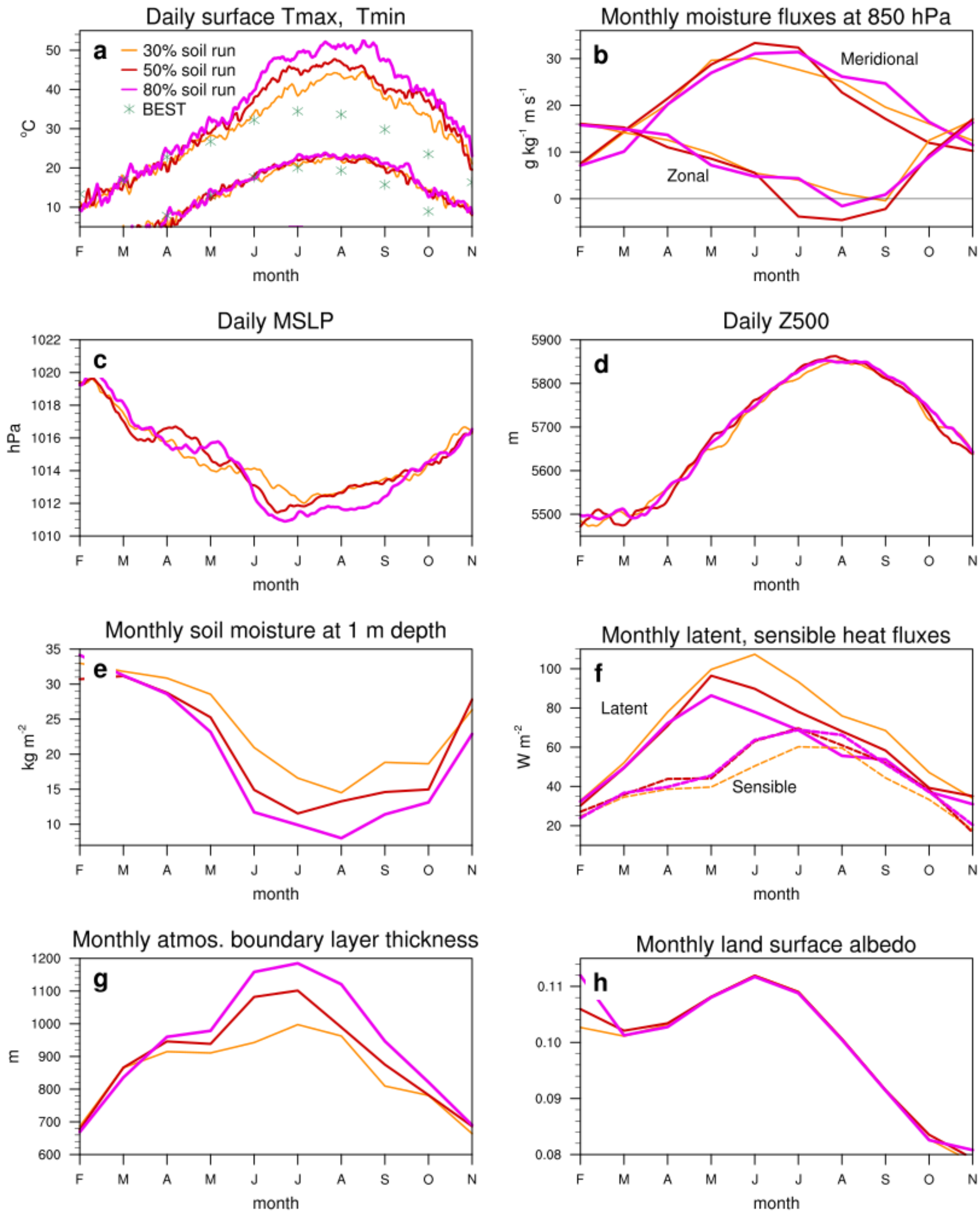

Supplementary Figure 8: Annual cycle of Dust Bowl surface and atmospheric conditions in bare soil experiments. Observed and simulated climatologies averaged over 1930-37 for the southern-central US (105°-85°W, 30°-40°N; a,b, e-h) and Midwest to eastern US (105°-75°W,

30°-50°N; c,d), for daily surface maximum and minimum temperature (Tmax, Tmin) (a), monthly zonal and meridional moisture fluxes at 850 hPa (b), daily mean sea level pressure (MSLP; c), daily 500 hPa geopotential height (Z500; d), monthly soil moisture at 1 m depth (e), monthly latent (solid) and sensible (dashed) heat fluxes (f), monthly atmospheric boundary layer thickness (g), and monthly land surface albedo (h). Observations in (a) are the Berkeley Earth Surface Temperature (BEST; asterisks) dataset, while simulations are the Hadley Centre Global Environment Model version 3 (HadGEM3) 30% (orange), 50% (red) and 80% (pink) soil runs.

Supplementary Reference

1. Worster, D. *Dust Bowl: The Southern Plains in the 1930s*. (Oxford Univ. Press, 1979).
